# Heterologous Prime Boost Vaccination Induces Protective Melanoma-Specific CD8^+^ T Cell Responses

**DOI:** 10.1016/j.omto.2020.10.001

**Published:** 2020-10-10

**Authors:** Sandra S. Ring, Michał Królik, Fabienne Hartmann, Erika Schmidt, Omar Hasan Ali, Burkhard Ludewig, Stefan Kochanek, Lukas Flatz

**Affiliations:** 1 Institute of Immunobiology, Kantonsspital St.Gallen, Rorschacher Strasse 95, 9007 St.Gallen, Switzerland; 2 Institute of Experimental Immunology, University of Zurich, Zurich, Switzerland; 3 Department of Gene Therapy, Ulm University, Helmholtzstrasse 8, 89081 Ulm, Germany; 4 Department of Dermatology, University Hospital Zurich, Gloriastrasse 31, 8091 Zurich, Switzerland; 5 Department of Oncology and Hematology, Kantonsspital St.Gallen, Rorschacher Strasse 95, 9007 St.Gallen, Switzerland; 6 Department of Dermatology, Kantonsspital St.Gallen, Rorschacher Strasse 95, 9007 St.Gallen, Switzerland

**Keywords:** Cancer vaccination, Viral Vectors, Melanoma, gp100, heterologous prime boost

## Abstract

Cancer vaccination aims at inducing an adaptive immune response against tumor-derived antigens. In this study, we utilize recombinant human adenovirus serotype 5 (rAd5) and recombinant lymphocytic choriomeningitis virus (rLCMV)-based vectors expressing the melanocyte differentiation antigen gp100. In contrast to single or homologous vaccination, a heterologous prime boost vaccination starting with a rAd5-gp100 prime immunization followed by a rLCMV-gp100 boost injection induces a high magnitude of polyfunctional gp100-specific CD8^+^ T cells. Our data indicate that an optimal T cell induction is dependent on the order and interval of the vaccinations. A prophylactic prime boost vaccination with rAd5- and rLCMV-gp100 protects mice from a B16.F10 melanoma challenge. In the therapeutic setting, combination of the vaccination with low-dose cyclophosphamide showed a synergistic effect and significantly delayed tumor growth. Our findings suggest that heterologous viral vector prime boost immunizations can mediate tumor control in a mouse melanoma model.

## Graphical Abstract

**Figure:**
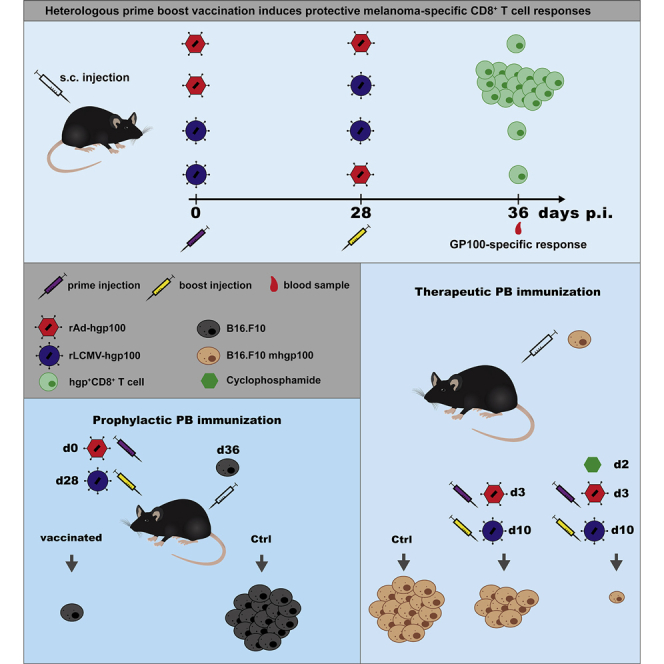

## Teaser

Virus-based vectors expressing a melanocyte differentiation antigen were used for a heterologous prime boost vaccination. Adeno prime immunization followed by a LCMV boost injection induced antigen-specific CD8^+^ T cells. Our findings suggest that heterologous viral vector prime boost immunizations can mediate tumor control in a mouse melanoma model.

## Introduction

Efficient vaccination against cancer and infectious diseases relies on the induction of adaptive immune responses. Cancer vaccines have successfully been applied for the prevention of cervical cancer and hepatocellular carcinoma by targeting human papillomavirus and hepatitis B virus, respectively.^1^^,^^2^ However, those vaccinations are preventive vaccines that target viral antigens of oncogenic viruses. To date there is no successful therapeutic cancer vaccine targeting shared self-antigens in patients with already existing tumors. Infiltration of cytotoxic CD8^+^ T cells into the tumor has been shown to play a key role in control and eradication of tumors.^3^ Current experimental vaccination protocols include peptides, cell-based vaccination, oncolytic viruses, or recombinant vectors.^4^^,^^5^ These approaches aim at activating adaptive immune responses through priming of naive T cells against the vector encoded antigen by professional antigen presenting cells (APCs). Vaccination vectors based on recombinant human adenovirus serotype 5 (rAd5) have extensively been studied in the context of T cell vaccines directed against viral antigens.^6^, ^7^, ^8^ Similarly, lymphocytic choriomeningitis virus (LCMV) has been shown to induce strong T cell responses by targeting dendritic cells without eliciting vector-specific antibodies and has recently been tested in a phase 1 clinical trial.^9^, ^10^, ^11^ To ensure safety, viral replication and dissemination can be restricted by genetic manipulation of viral vectors. Replication-deficient Ad5 vectors are generated by substituting the early transcribed E1 and/or E3 regions with a transgene or vaccine antigen. Absence of E1 in recombinant Ad (rAd) vectors limits replication within target cells.^12^ Recombinant lymphocytic choriomeningitis virus (rLCMV) was generated by reverse genetic engineering, i.e., the glycoprotein (GP) encoding sequence was replaced with a target antigen rendering the prototypic arenavirus propagation deficient. Induction of potent antitumoral T cells depends on the identification of the appropriate tumor-associated antigen provided by the viral vector. While it has been shown that murine gp100 (mgp100) fails to elicit a T cell response, cross-reactive human gp100 (hgp100) is able to induce antigen specific CD8^+^ T cells *in vivo.*^13^ Here, we show that heterologous prime boost (PB) immunization with rAd5- and rLCMV- expressing the melanoma-associated antigen hgp100 induces highly functional CD8^+^ T cells specific for hgp100 and mgp100. This results in a prophylactic protection from B16.F10 melanoma growth and transient tumor control in a therapeutic setting. Moreover, combination with low-dose cyclophosphamide (CTX) synergistically enhances tumor control and decelerates tumor growth in PB vaccinated mice.

## Results

### Single Immunization with Either rAd or rLCMV Vectors Induces Low Frequencies of hgp100-Specific CD8^+^ T Cells

**Figure 1:**
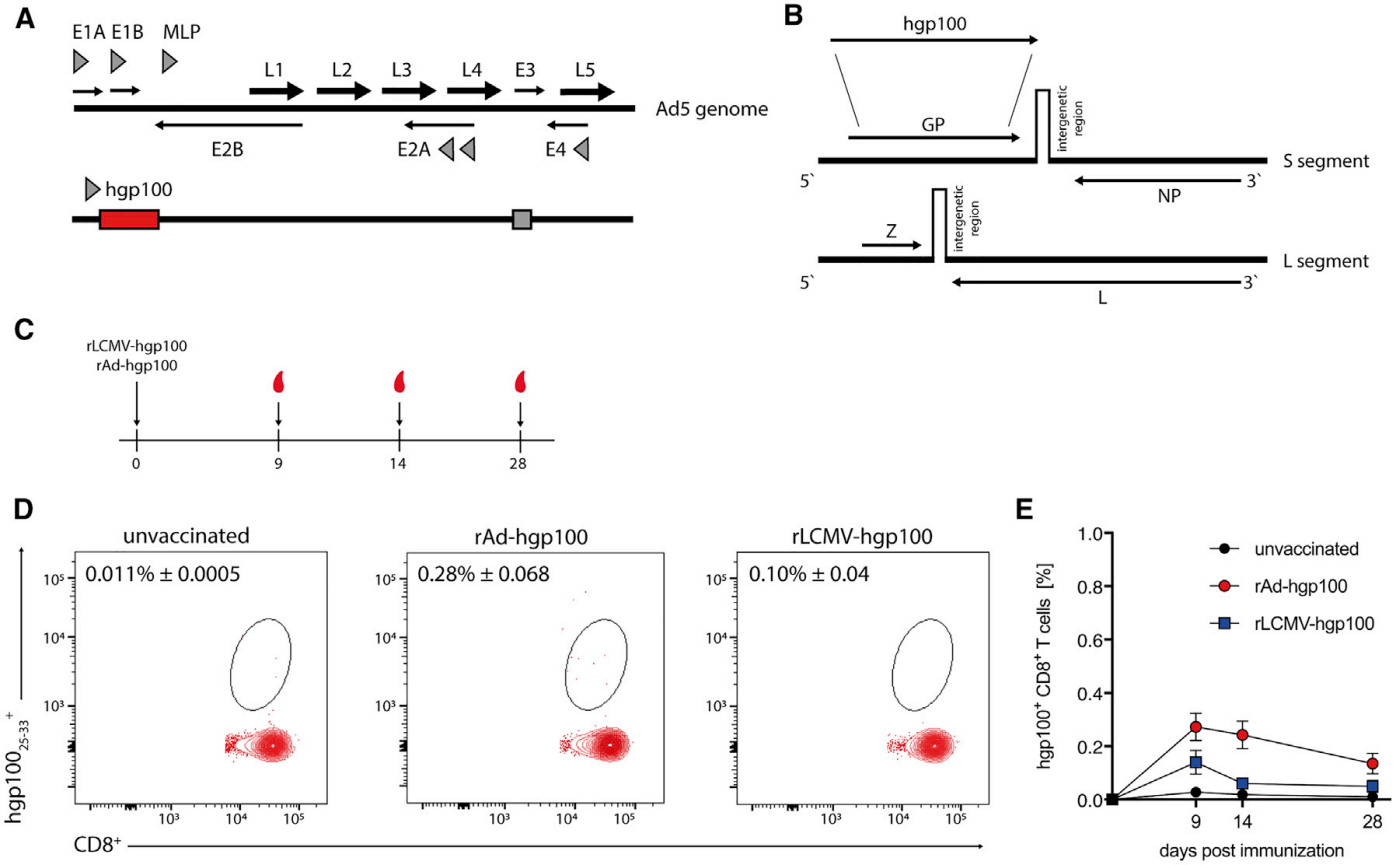
rLCMV Vectors Induce Low Frequencies of hgp100^+^ CD8^+^ T Cells (A) Linear, double-stranded DNA genome of wild-type HAdV-5 and the rAd5 vector genome. The adenoviral E1 genes are replaced with the hgp100 expression cassette. Figure modified from Volpers and Kochanek.^14^ (B) LCMV genome comprising of two RNA segments (S and L). The GP ORF is replaced by the full-length hgp100 antigen in rLCMV vectors. Figure modified from Ring and Flatz.^15^ (C) Vaccination scheme for subcutaneous immunization of C57BL/6 mice with either rAd or rLCMV expressing hgp100. Sampling of blood at day 9, 14, and 28 post immunization. (D and E) Representative FACS blots (D) and frequencies (E) of CD8^+^ T cells specific to the H-2D^b^ restricted hgp100_25–33_ epitope measured in peripheral blood of immunized C57BL/6 mice day 9, 14, and 28 post immunization. Data are pooled from three independent experiments with n = 3–8 mice per group. ∗p < 0.5, ∗∗p < 0.01, ∗∗∗p < 0.001.

We utilized the genetically modified rAd5 and reverse genetically engineered rLCMV for the immunization of C57BL/6 mice expressing the full-length hgp100 (Figures 1A and 1B). hgp100 shows an enhanced binding to H-2Db compared to mgp100 improving epitope presentation. However, mgp100 was reported to be sufficient for recognition of cross-reactive CD8^+^ T cells.^13^ After single subcutaneous immunization with either rAd-hgp100 or rLCMV-hgp100 we analyzed hgp100-specific CD8^+^ T cell kinetics in peripheral blood at the indicated time points post immunization (Figure 1C). We observed that single injections of rAd-hgp100 or rLCMV-hgp100 both failed to induce a significant increase of hgp100_25–33_-specific CD8^+^ T cells compared to unvaccinated controls (Figures 1D and 1E). Hence, single injection of Ad or LCMV vectors with hgp100 does not induce a strong adaptive immune response.

#### Heterologous Prime Boost Immunization with rAd and rLCMV Boosts hgp100^+^ T Cell Response

**Figure 2:**
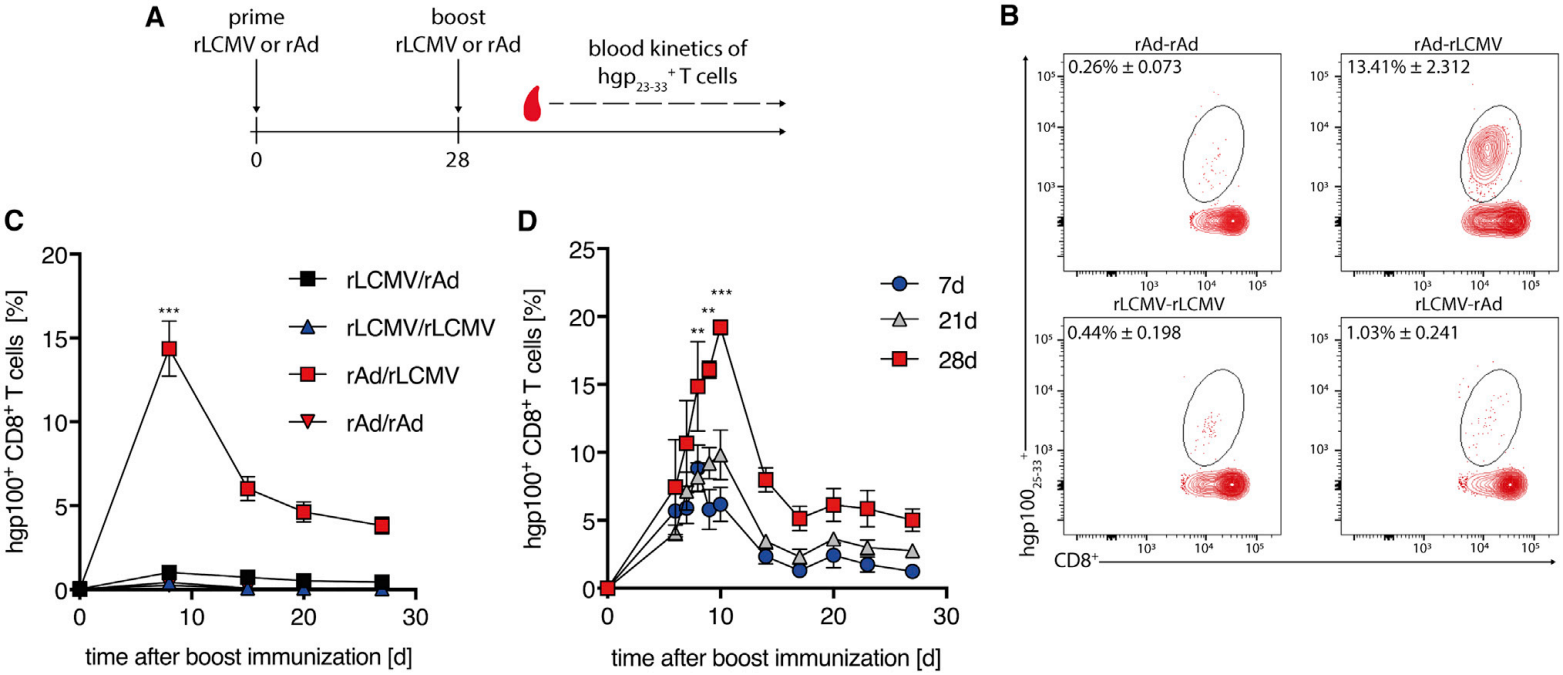
Heterologous Prime Boost Immunization with rAd and rLCMV Improves hgp100^+^ T Cell Response (A) At day 0 mice were immunized s.c. with rAd- or rLCMV-hgp 100. On day 28 mice received a homologous or heterologous boost immunization with the respective vectors. (B) Representative FACS plots gated on CD8^+^ T cells specific to the H-2D^b^ restricted hgp100_25–33_ epitope. Mice received an injection of rAd and rLCMV-hgp100 in different PB combinations. (C) Frequencies of hgp100^+^ CD8^+^ T cells measured in peripheral blood of immunized C57BL/6 mice at indicated time points after boost immunization. Pooled data represent the mean ± SEM of three independent experiments with n = 4–5 mice per group. (D) Frequencies of CD8^+^ T cells specific to the hgp100_25–33_ epitope measured in peripheral blood of prime-boost immunized C57BL/6 mice. Mice received prime immunization with rAd5 and boost immunization with rLCMV expressing the hgp100 antigen in a different time interval. Data are pooled from 2 independent experiments with n = 4–5 mice per group. ∗p < 0.5, ∗∗p < 0.01, ∗∗∗p < 0.001.

In order to increase the frequency of hgp100-specific CD8^+^ T cells, we performed PB immunization strategies. For this purpose, we used either vector for prime- and boost immunization expressing the identical hgp100 and assessed the frequencies of hgp100 specific CD8^+^ T cells in peripheral blood of mice at the indicated time points (Figure 2A). Homologous PB of either vector had no significant impact on the induction of hgp100-specific CD8^+^ T cells. However, when we used heterologous PB protocols, we were able to measure a significant increase of hgp100 specific CD8^+^ T cells in peripheral blood. Interestingly, the order of the vectors used had a crucial impact on eliciting hgp100-specific CD8^+^ T cells with rAd prime and rLCMV boost immunization demonstrating the highest hgp100-specific CD8^+^ T cell responses. Responses in mice primed with rAd-hgp100 and boosted with rLCMV-hgp100 peaked at 8 days after boost immunization (Figures 2B and 2C). To investigate the importance of the interval between rAd-hgp100 prime and rLCMV-hgp100 boost immunization, we reduced the time interval between the prime and the boost immunization from 4 weeks to 3 or 1 week, respectively, confirming the dependence on the time interval to induce those high CD8^+^ T cell responses. The highest frequencies of hgp100 specific CD8^+^ T cells were observed when rLCMV-hgp100 was injected 28 days after rAd-hgp100 prime immunization (Figure 2D). These data demonstrate the feasibility of a heterologous PB immunization for T cell priming directed against gp100. Furthermore, optimization experiments revealed that rAd prime followed by a rLCMV boost with a 4-week interval yielded the most potent T cell response.

#### Heterologous PB Immunization with rAd and rLCMV Induces Polyfunctional CD8^+^ T Cells

**Figure 3:**
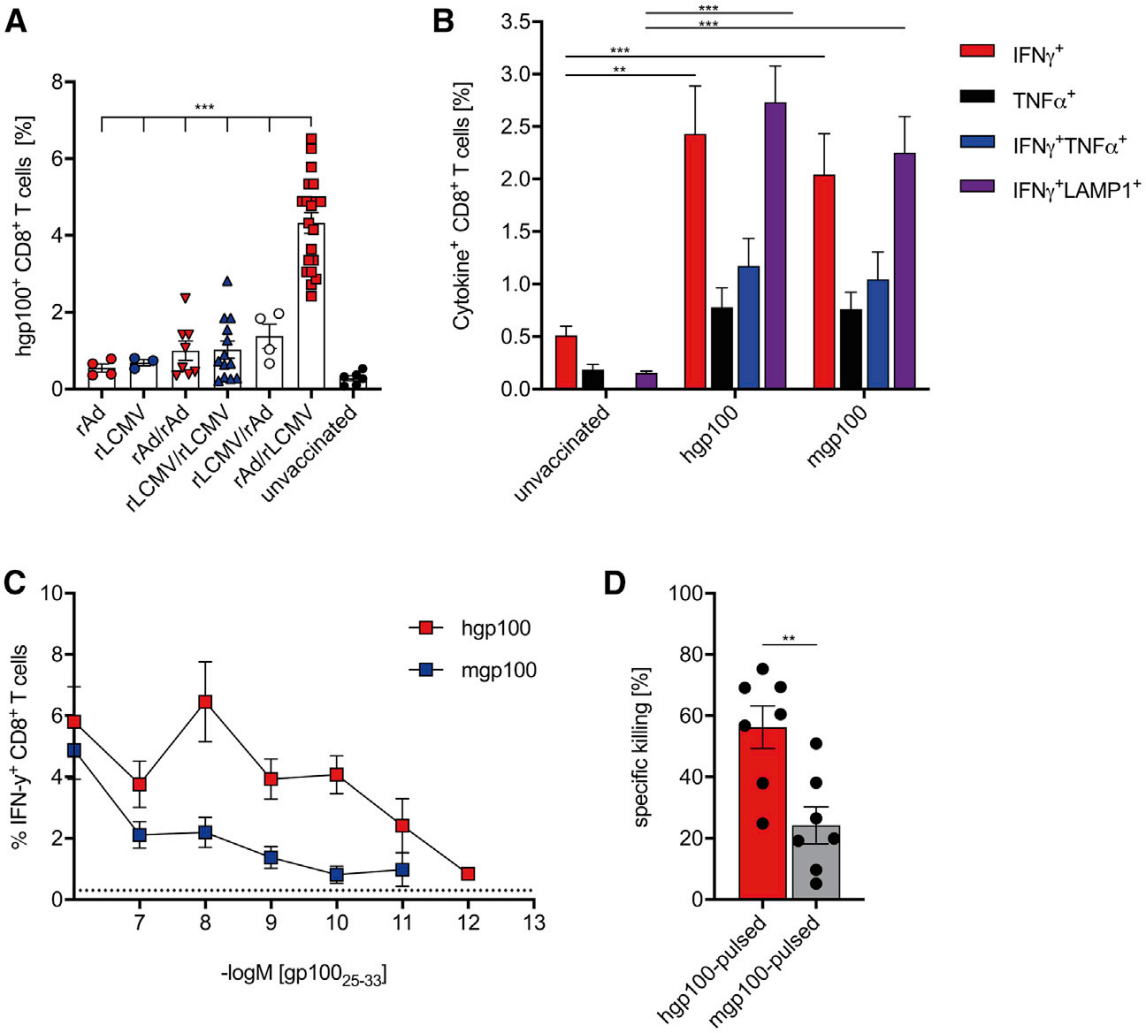
Heterologous Prime Boost Immunization with rAd/rLCMV Induces Polyfunctional CD8^+^ T Cells (A) Frequencies of hgp100-specific CD8^+^ T cells in the spleen of immunized C57BL/6 mice. Mice received a rAd-hgp100 prime and rLCMV-hgp100 boost immunization 28 days later. Frequencies of hgp100^+^ CD8^+^ T cells 8 days post boost immunization measured in the spleen. Pooled data from four independent experiments with n = 4–6 mice per group. (B) Frequencies of cytokine (IFN-γ, TNF-α)-producing and LAMP1^+^ (CD107a^+^) expressing CD8^+^ T cells after *in-vitro* re-stimulation of splenocytes with the hgp100_25–33_ or mgp_25–33_ peptide from five independent experiments with n = 3–6 mice per group. (C) Frequencies of IFN-γ-producing CD8^+^ T cells after re-stimulation of splenocytes with different concentrations of hgp100_25–33_ or mgp100_25–33_ peptide. Data was pooled from five independent experiments with n = 3–5 mice per group. (D) Prime-boost immunized mice were intravenously injected with either hgp100_25–33_ or mgp100_25–33_ peptide pulsed splenocytes. 24 h later the CFSE expression in peripheral blood was analyzed and the specific killing of peptide pulsed cells determined. Data was pooled from two independent experiments with n = 3–4 mice per group. ∗p < 0.5, ∗∗p < 0.01, ∗∗∗p < 0.001.

To elaborate on the functionality of vaccine-induced hgp100-specific CD8^+^ T cells, we assessed their frequency in the spleen (Figure 3A). At day 8 after PB vaccination we observed significantly increased hgp100-specific CD8^+^ T cells in the spleen of immunized mice compared to unvaccinated control animals. To determine the potential of the hgp100-induced CD8^+^ T cells to produce cytokines, we re-stimulated splenocytes *ex vivo* with the hgp100 or the mgp100 peptide to demonstrate their cross-reactivity.

We found that CD8^+^ T cells from heterologous rAd/rLCMV vaccinated mice were polyfunctional demonstrated by a significantly increased production of interferon-γ (IFN-γ), tumor necrosis factor alpha (TNF-α), and CD107a (LAMP1) compared to naive controls (Figure 3B). As hypothesized, vaccination with hgp100-expressing viral vectors gave rise to cross-reactive CD8^+^ T cells recognizing murine gp100 and producing IFN-γ upon re-stimulation *ex vivo*. Cytokine production in CD8^+^ T cells was increased when re-stimulated with hgp100 compared to equal amounts of mgp100, confirming an enhanced binding affinity of hgp100 to H2Db molecules (Figure 3C), as has been previously shown.^13^ For measurement of CTL activity and determination of cell-mediated cytotoxicity, we examined the specific *in vivo* killing ability of CD8^+^ T cells derived from rAd/rLCMV PB immunized mice. Injection of vaccinated mice with Carboxyfluorescein succinimidyl ester (CFSE)-labeled splenocytes loaded with the respective hgp100 or mgp100 peptide resulted in the elimination of the transferred splenocytes. Remarkably, specific killing was 3-fold higher against hgp100 pulsed target cells compared to mgp100 target cells (Figure 3D). These results suggest that PB with hgp100 expressing rAd and rLCMV vectors induce high frequencies of cross-reactive hgp100 and mgp100 specific CD8^+^ T cells that are characterized by high functionality and cytotoxicity.

#### Protection from B16.F10 Challenge by Heterologous Prime Boost Immunization with rAd/rLCMV

Since heterologous rAd/rLCMV PB immunization significantly increased hgp100-specific CD8^+^ T cell frequencies, we sought to investigate whether they were proficient to protect mice from a B16.F10 melanoma challenge.

**Figure 4:**
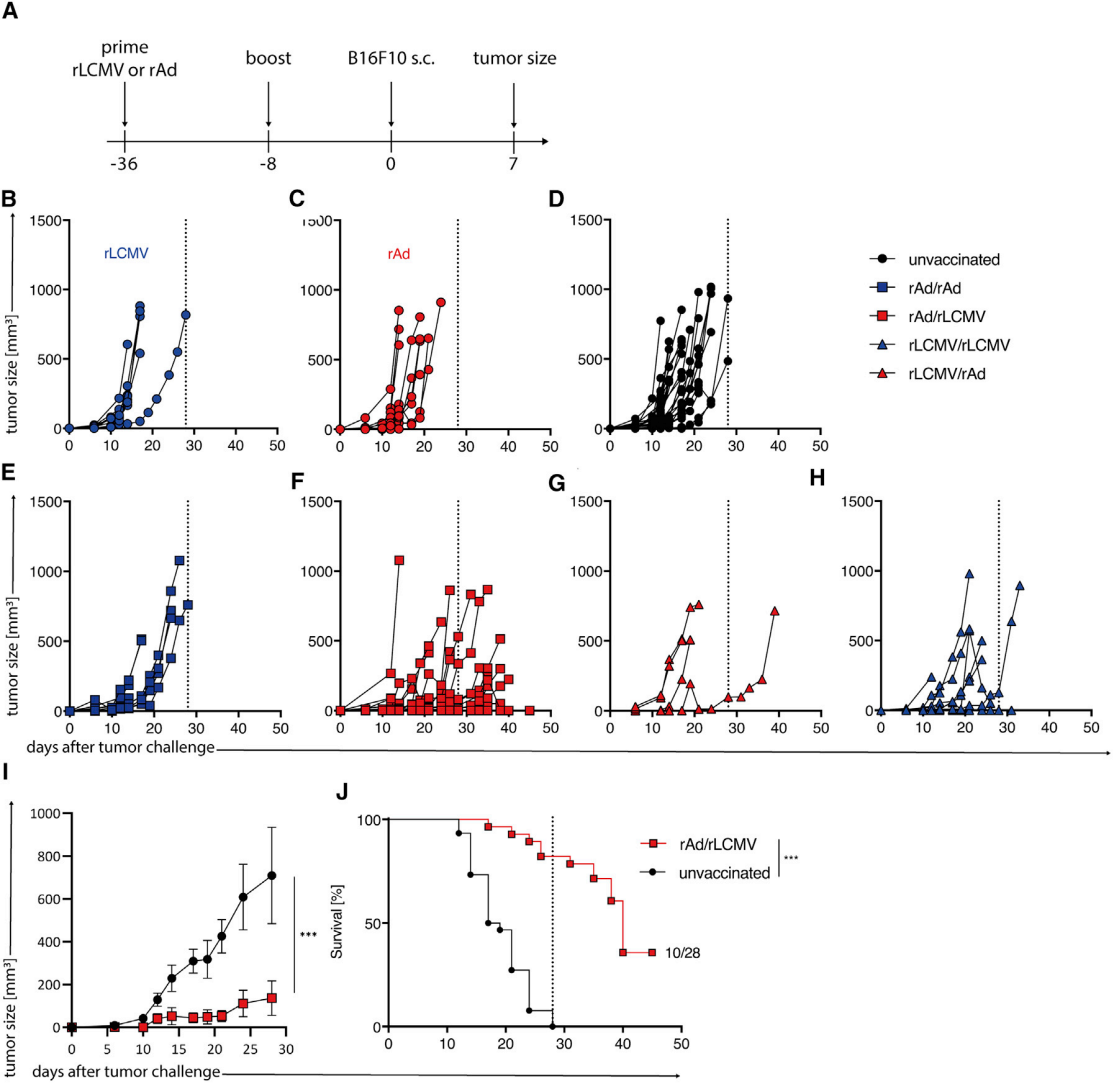
Heterologous Prime Boost Immunization Limits C57BL/6 Mice from B16.F10 Mouse Melanoma Tumor Growth (A) Experimental setup scheme for prophylactic PB immunization of B16.F10 challenged C57BL/6 mice. Mice received a s.c. injection of rAd-hgp100 or rLCMV-hgp100. 28 days later, mice were boost-immunized as indicated. At day 7 after boost immunization mice were challenged with s.c. injection of 1 × 10^5^ cells B16.F10 and (B–H) tumor growth was monitored. (I) Tumor size of mice PB immunized with rAd-rLCMV both expressing hgp100. (J) Survival of rAd/rLCMV immunized mice compared to unvaccinated control mice. Indicated long-term survivors out of included mice. ∗p < 0.5, ∗∗p < 0.01, ∗∗∗p < 0.001.

We immunized mice subcutaneously (s.c.) with rAd- and rLCMV-hgp100 as indicated and challenged them with B16.F10 melanoma cells by s.c. inoculation at day 8 after the boost immunization (Figure 4A). Single vaccination with either rAd or rLCMV vectors expressing hgp100 had no impact on B16.F10 tumor growth compared to unvaccinated mice (Figures 4B and 4C). In line with our immunogenicity data, only rAd-hgp100 priming followed by rLCMV-hgp100 boosting had a significant impact on tumors, which were either strongly impaired in growth or prevented from growing (Figures 4D–4H). In contrast, homologous PB or rLCMV priming followed by a rAd boost did not show any effect (Figure 4I). These data suggest that a PB vaccination with rAd/rLCMV acts as a potent prophylactic vaccine regimen in mice protecting them from a B16.F10 tumor challenge.

#### Therapeutic PB Immunization with hgp100 Is More Efficient against mhgp100 Expressing Melanoma and Enhanced by CTX

**Figure 5:**
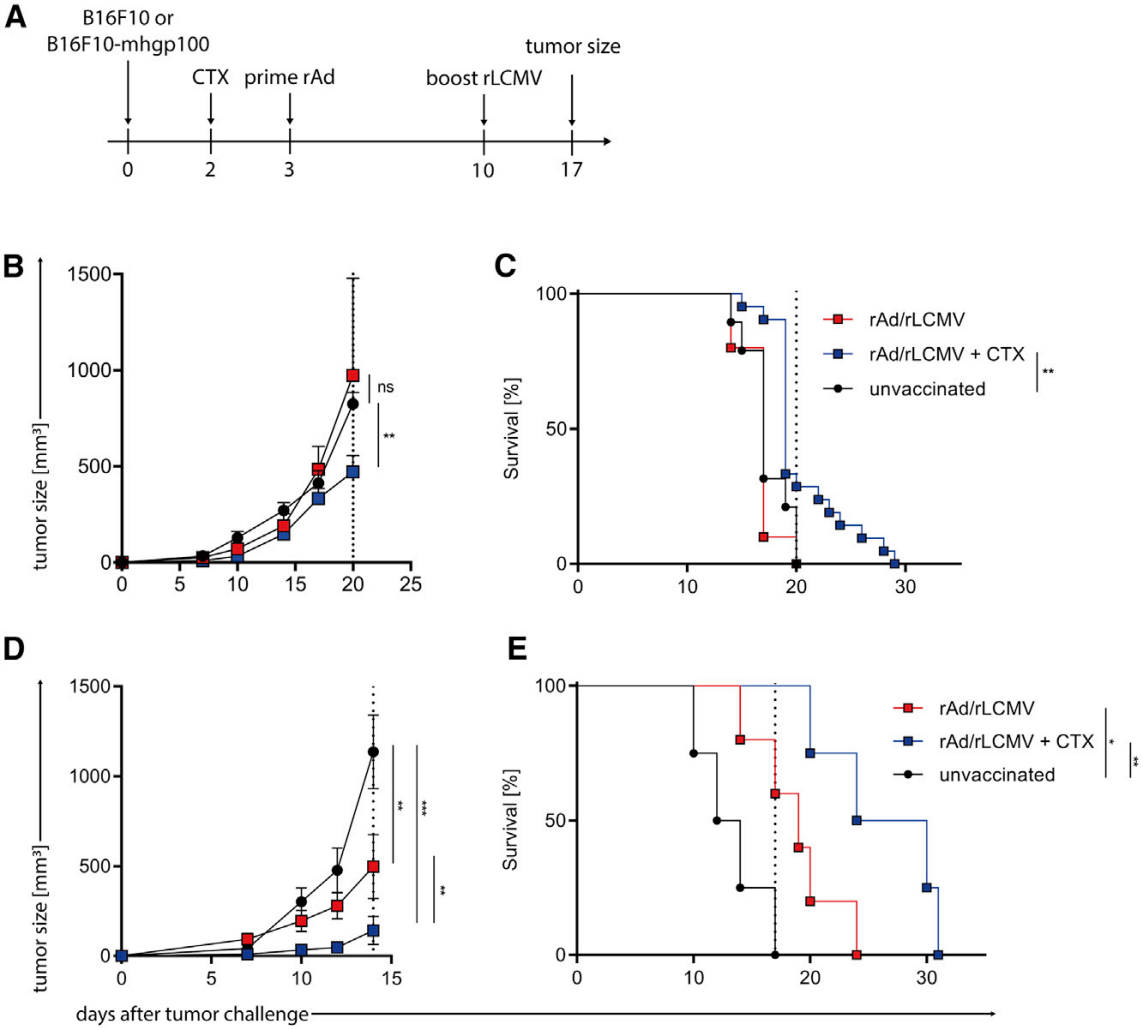
Therapeutic Immunization with hgp100 against mhgp100 Expressing Melanoma Is Enhanced by CTX (A) Experimental setup scheme for therapeutic PB immunization of C57BL/6 mice. Mice were inoculated s.c. with 1 × 10^5^ cells B16.F10. After 2 days mice were treated intraperitoneally (i.p.) with cyclophosphamide (CTX). At day 3 mice were subcutaneously prime-immunized with 1 × 10^9^ particles rAd-hgp100. At day 10 mice received a boost vaccination with rLCMV-hgp100. (B and C) Tumor growth kinetics in mice prime boost immunized with rAd/rLCMV-hgp100 or rAd/rLCMV-hgp100 in combination with CTX compared to untreated control mice (B) and corresponding survival of mice (C). (D and E) Growth kinetics (D) and survival (E) of B16.F10 expressing mhgp100 in mice immunized with rAd/rLCMV-hgp100 or rAd/rLCMV-hgp100 in combination with CTX compared to untreated control mice. ∗p < 0.5, ∗∗p < 0.01, ∗∗∗p < 0.001.

To assess the feasibility of rAd/rLCMV PB for a therapeutic vaccination, we utilized B16.F10 tumor-bearing mice and injected rAd-hgp100 3 days and a rLCMV-hgp100 boost 10 days after tumor challenge (Figure 5A). Therapeutic vaccination had no significant impact on tumor size compared to unvaccinated mice, most likely due to the fast-growing tumor and the limited time for the development of an adaptive immune response due to the short prime boost interval. As it has previously been shown that melanoma patients receiving adoptive cell therapy benefit from low-dose CTX, we investigated whether the additional application of low-dose CTX is capable of improving the heterologous PB vaccination.^16^, ^17^, ^18^ Accordingly, 1 day prior to rAd/rLCMV PB immunization, we treated mice with 200 μg CTX. This led to significantly delayed tumor growth and indicates a synergistic effect between CTX and the PB immunization (Figure 5B). CTX application alone did not result in an anti-tumor effect (Figures S1A and S1B). In line with these findings, the additional application of CTX resulted in a significant prolongation of survival (Figure 5C). We then proceeded to evaluate whether full tumor remission can be achieved without CTX using a B16.F10 melanoma cell line expressing mhgp100 (B16.F10-mghp100), a chimeric antigen with neoantigen properties that increases the immunogenicity of the tumor.^19^^,^^20^ Indeed, mice challenged with B16.F10-mhgp100 receiving PB vaccination showed decreased tumor growth (Figure 5D) and prolonged survival even without the application of CTX (Figure 5E). Our results in the B16.F10 melanoma model demonstrate the feasibility of a therapeutic prime boost vaccination directed against the public melanocyte differentiation antigen gp100 without the need of an adoptive T cell transfer. Furthermore, the prime boost vaccination synergizes with the application of low dose CTX.

## Discussion

Vaccinations against oncogenic viruses including hepatitis B and human papilloma virus have significantly decreased the incidence of hepatocellular and cervical cancer.^1^^,^^2^ However, therapeutic cancer vaccines targeting self-antigens have not yet been FDA-approved due to poor results in clinical trials.^21^ gp100 is a classical immunogenic melanocyte differentiation antigen that has been studied for decades.^13^^,^^22^, ^23^, ^24^ Cancer vaccines targeting gp100 particularly in combination with recombinant interleukin-2 (IL-2) resulted in some benefit for melanoma patients.^18^^,^^22^ Moreover, adoptive transfer of *in vitro* expanded tumor-infiltrating lymphocytes (TILs) directed against gp100 induced tumor regression in several patients.^16^ Furthermore, extensive studies in the B16.F10 mouse melanoma model using adoptive T cell transfer and vaccination targeting gp100 have demonstrated tumor control.^20^^,^^25^, ^26^, ^27^, ^28^ Of note, many melanoma patients lack a high number of TILs, indicating the need for TIL-inducing vaccines. Ad-based vectors are frequently used antigen delivery platforms for vaccines in clinical studies, as they have been shown to be potent inducers of specific T cell responses.^12^^,^^29^^,^^30^ Yet, vaccination with melanoma-associated antigen expressing rAd vectors alone failed to successfully decrease tumor burden in the mouse melanoma models. Homologous PB vaccination commonly leads to the development of virus-specific adaptive immune responses,^31^^,^^32^ severely limiting their suitability to induce a sustainable adaptive immune response. Our heterologous PB vaccination circumvents this problem by using two fundamentally different virus classes: while rAd is based on a non-enveloped double-stranded DNA virus, rLCMV originates from an enveloped negative-strand RNA virus. This allows the presentation of the same antigen in the context of two different PAMPS (pathogen-associated molecular patterns) activating different pattern recognition receptors.^9^^,^^15^ Indeed, the heterologous vaccination strategy leads to a significantly increased number of gp100-specific T cells. Surprisingly, the sequence and interval of the vector application is of critical importance. A similar phenomenon has already been shown using a viral antigen.^33^ This could be due to different effector T cell populations induced by the first vaccination.^34^ While PB immunization strategies have been applied for therapeutic vaccination,^35^, ^36^, ^37^, ^38^, ^39^ our attempt to utilize a novel protocol of heterologous PB vaccination for therapeutic immunization showed insufficient control over B16.F10 growth without addition of CTX. Cancer specific mutations of self-antigens result in tumor specific neoantigens enhancing tumor immunogenicity. Based on a previously described mouse melanoma model,^20^ we investigated the therapeutic effect of the vaccination by inoculating mice with a genetically modified B16.F10 melanoma cell line that expresses chimeric mhgp100 as a model for a neoantigen. Vaccinated mice were able to control this more immunogenic B16.F10-mhgp100 tumors indicating a suitability of the PB protocol for neoantigens. Low-dose CTX has demonstrated unique immune-modulating effects that can be exploited for indirect targeting of tumors.^40^ Along with the upregulation of proinflammatory cytokine/chemokine production and T helper 1 (Th1)/Th17 responses, it has been shown that low dose CTX can reduce levels of regulatory T cells (T_reg_) resulting in a beneficial milieu for T cells favoring tumor eradication.^41^^,^^42^ Additional studies reported a transient depletion of bone marrow cells by CTX and impairment of myelopoiesis ultimately affecting a vaccination protocol strongly relying on APC-mediated effector T cell activation and priming.^43^, ^44^, ^45^ Furthermore, there are reports indicating type-I IFN secretion driving DC maturation and counteracting the perturbated myeloid cell homeostasis.^46^ Beneficial effects of CTX on cancer immunotherapy and by a co-administration with a vaccine have been demonstrated in pre-clinical mouse models.^47^^,^^48^ However, clinical studies with a different dosing of CTX and limited mechanistical insight prompt additional studies of CTX on the human immune system.^49^, ^50^, ^51^, ^52^, ^53^

In conclusion, our data demonstrate that PB immunization using rAd and rLCMV vectors can induce CD8^+^ T cells and their specific killing *in vivo* but fall short of therapeutic tumor control and elimination when wild-type self-antigens are targeted. Tumor expression of immunogenic neo-antigens might overcome this limitation and may potentially lead to an effective vaccination with a durable response.

## Material and Methods

### Cells and Cell Lines

Murine B16.F10 cells were obtained from the American Type Culture Collection and cultured in Dulbecco’s modified Eagle’s medium (DMEM, GIBCO, Buchs, Switzerland) supplemented with 10% (vol/vol) fetal calf serum (FCS) (Sigma-Aldrich, St. Louis, MO, USA), 10 mmol/L NEAAs (GIBCO, Buchs, Switzerland), 1 mmol/L sodium pyruvate (GIBCO, Buchs, Switzerland), 100 IU/mL penicillin/streptomycin (Lonza, Basel, Switzerland). B16.F10-mhgp100 were a gift from Nicholas P. Restifo (National Cancer Institute, Bethesda, MD, USA). BHK-21GPtg and HEK293GPtg cells for viral vector production were obtained from the Institute of Experimental Immunology, University of Zurich. All cell lines were kept at 37°C and 5% CO_2_ in a humidified incubator and regularly examined for mycoplasma.

### Animals and Housing

6- to 8-week-old C57BL/6 mice were purchased from Charles River (Sulzfeld, Germany). For experiments, sex- and age-matched mice between 7 and 10 weeks of age were used. Experiments were performed in accordance with federal and cantonal guidelines (Animal Welfare Act) following review and approval by the Cantonal Veterinary Office of St. Gallen, Switzerland (approval number SG03/18). All mice used for experiments were housed in the animal care facility at the Institute for Immunobiology Saint Gallen according to the required biosafety level for SPF mice.

### Viral Vectors

Propagation-deficient rLCMV expressing hgp100 (rLCMV-hgp100) was generated using reverse genetic cDNA technology and the hgp100 sequence was inserted into Pol-I-Sv. For the rescue of recombinant LCMV vectors, a previously described four plasmid co-transfection system was used.^9^ High-titer stocks of 1 × 10^7^ particles per mL were stored at −80°C. An E1-deleted Ad5 vector expressing hgp100 was generated as described.^54^ Briefly, an expression cassette expressing full-length hgp100 under control of the hCMV promotor was inserted into the PacI site of pGS66.^54^ The plasmid DNA was cleaved with SwaI and the vector was produced in N52.E6 cells as described,^54^ followed by purification of CsCl density ultracentrifugation and determination of the physical particle (pp) titer.^14^^,^^55^

### Immunization and Tumor Challenge of Experimental Mice

Experimental mice were injected with 1 × 10^9^ pps of rAd-hgp100 in PBS or 5 × 10^5^ pfu rLCMV-hgp100 in sterile DMEM (GIBCO, Buchs, Switzerland). Immunizations were carried out by subcutaneous injection into the flank. Mice were subcutaneously inoculated into the flank with 1 × 10^5^ B16.F10 for the mouse melanoma model. Tumor measurements were started around 7 days post tumor challenge when tumors became palpable. The experimental mice were euthanized when the tumors exceeded 1,000 mm^3^ or when defined endpoint criteria were reached.

### Blood Kinetics of CD8^+^ T Cells Using Fluorescence-Activated Cell Sorting (FACS)

Blood was sampled in a FACS tube containing 3 mL ice cold 1× FACS Buffer. The sample was centrifuged at 1,200 rpm for 5 min at 4°C. Cells were resuspended in 1× FACS Buffer containing H2Kb hgp100-PE multimer (1:100 diluted) and incubated at 37°C for 10 min. After incubation cells were washed and utilized for surface staining on ice for 20 min. Afterward the sample was washed with 1× FACS Buffer and centrifuged at 1,200 rpm for 5 min at 4°C. The cells were resuspended in 0.5 mL BD Lysis buffer for erythrocyte lysis and immediately vortexed. The cells were incubated for 5 min at room temperature (RT) and washed with 1× FACS Buffer. Cells were then centrifuged and resuspended in 100 μL FACS Buffer. Samples were subjected for measurement of antigen-specific CD8^+^ T cell frequencies using FACS Canto II.

### Analysis of CD8^+^ T Cell Functionality with the Intracellular Cytokine Staining Assay

Mice were either prime immunized with 1 × 10^9^ pp rAd-hgp100 and 1 to 5 × 10^5^ pfu rLCMV-hgp100. The mice were sacrificed either at day 9 post prime immunization or 8 days post boost immunization. The mouse was dissected and the spleen isolated. The fresh spleen was added to a 15 mL Falcon Tube containing RPMI-1640 (GIBCO, Buchs, Switzerland) supplemented with 5% FCS (GIBCO, Buchs, Switzerland) and placed on ice. The spleen was mechanically disrupted within a Petri dish using a 70 μm cell strainer (Falcon, Corning) and a syringe plunger. The single cell suspension was collected in a 15 mL Falcon tube and centrifuged at 1,200 rpm for 5 min at 4°C. Cells were resuspended in RPMI-1640 containing 5% FCS. 100 μL cell suspension was used for staining of tetramer-specific CD8^+^ T cells. PMA/Ionomycin (P/I; Sigma-Aldrich, Buchs, Switzerland) was used as positive control. The exocytose blocker Brefeldin A (Sigma-Aldrich, Buchs, Switzerland) was used to block cytokine secretion thus to enable the intracellular cytokine staining. Brefeldin A was diluted in RPMI-1640 to a final concentration of 10 μg/mL. Single cell suspended cells were seeded in a round-bottom 96 well plate. Controls and peptides were added to the samples and after addition of Brefeldin A incubated at 37°C for 5 h. After 5 h the cells were washed with 1× FACS Buffer and resuspended in 1× FACS Buffer containing anti-mouse CD8a-APC (1:100). Cells were incubated at 4°C for 30 min. After washing cells were resuspended in 100 μL Cytofix/Cytoperm (BD Bioscience, Allschwil, Switzerland) solution and incubated at 4°C for 20 min. To permeabilize cells Permeabilization Buffer (PB; Invitrogen, Dietikon, Switzerland) was added to wash the cells. Cells resuspended in PB containing anti-mouse IFN-γ-PE and TNF-α-fluorescein isothiocyanate (FITC; 1:50 diluted; Biolegend, San Diego, CA, USA) and incubated at 4°C for 40 min. Cells were washed with PB twice, resuspended in 1× FACS Buffer, and measured by FACS Canto II.

### *In Vivo* Cytotoxicity Assay

In order to investigate the *in vivo* cytotoxicity, immunized mice were intravenously injected with either human or murine gp100 pulsed target cells on day 8 after boost immunization. Shortly after erythrocyte lysis via osmotic shock, single cell suspended cells were incubated with 10^−6^ M hgp100_25–33_ or mgp100_25–33_ for 1.5 h at 37°C or left untreated. Cells were labeled using 10 μL 5 mM CFSE for pulsed or 0.5 mM CFSE for unpulsed cells according to the manufacturer’s protocol (CellTrace CFSE Cell Proliferation Kit Protocol; Invitrogen). Pulsed and unpulsed splenocytes were mixed 1:1 and 3–5 × 10^7^ cells and intravenously injected into vaccinated mice. 24 h after transfer, CFSE expression in blood was analyzed and specific killing was calculated using the following formula 100−([(% peptide-pulsed in infected/% unpulsed in infected)/(% peptide-pulsed in uninfected/% unpulsed in uninfected)] × 100).

### Statistical Analysis

Statistical analysis was performed using GraphPad 8.0. Unless specified otherwise, graphs depict mean ± SEM. Differences between two groups were evaluated using unpaired two-tailed Student’s t tests. Single values of multiple groups were compared with two-way analysis of variance (ANOVA) followed by Bonferroni post hoc test. Kaplan Meier Survival curves were assessed using log-rank test. Results were considered statistically significant when ∗p < 0.05, ∗∗p < 0.01, and ∗∗∗p < 0.001.

## Author Contributions

S.S.R., M.K., and L.F. designed the study. S.S.R., F.H., E.S., S.K., and L.F. developed the methodology and S.S.R. and F.H. acquired data. The analysis and interpretation of data (e.g., statistical analysis, biostatistics, computational analysis) was conducted by M.K., S.S.R. and L.F. M.K. and L.F. wrote the manuscript. S.S.R., O.H.A., and B.L. helped review the manuscript. E.S., S.K., and L.F. contributed with administrative and technical support and supported the study with material. L.F. supervised the study. All authors read and approved the final manuscript.

## Conflicts of Interest

The authors declare no competing interests.

## Supplemental Information

Document S1. Figures S1–S3

Document S2. Article plus Supplemental Information
