# Supplementary material for: Heterologous Prime Boost Vaccination Induces Protective Melanoma-Specific CD8+ T Cell Responses
Source: Mol Ther Oncolytics. 2020 Oct 10;19:179–87. doi: 10.1016/j.omto.2020.10.001 (PMC7658660; doi:10.1016/j.omto.2020.10.001)
Supplement: Document S1. Figures S1–S3 [file mmc1.pdf]

OMTO, Volume *19*

## Supplemental Information

# Heterologous Prime Boost Vaccination Induces Protective Melanoma-Specific $CD8^+$ T Cell Responses

**Sandra S. Ring, Michał Królik, Fabienne Hartmann, Erika Schmidt, Omar Hasan Ali, Burkhard Ludewig, Stefan Kochanek, and Lukas Flatz**

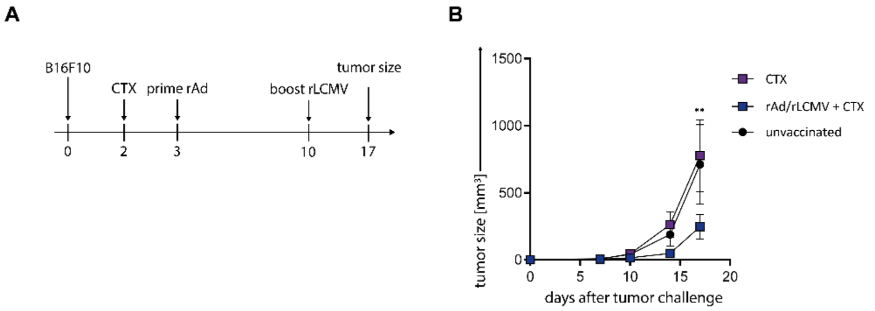


**Supplement Fig. 1 Therapeutic immunization with hgp100 against B16.F10 is enhanced by CTX. A** Experimental setup scheme for therapeutic PB immunization of C57BL/6 mice. Mice were inoculated s.c. with $1x10^5$ cells B16.F10. After two days mice were treated i.p. with CTX. At day 3 mice were subcutaneously prime-immunized with $1x10^9$ particles rAd-hgp100. At day 10 mice received a boost-vaccination with rLCMV-hgp100 **B** Tumor growth kinetics in mice prime boost immunized with rAd/rLCMV-hgp100 or rAd/rLCMV-hgp100 in combination with CTX compared to CTX treated mice and untreated control mice. **P* < 0.5, ***P* < 0.01, ****P* < 0.001.

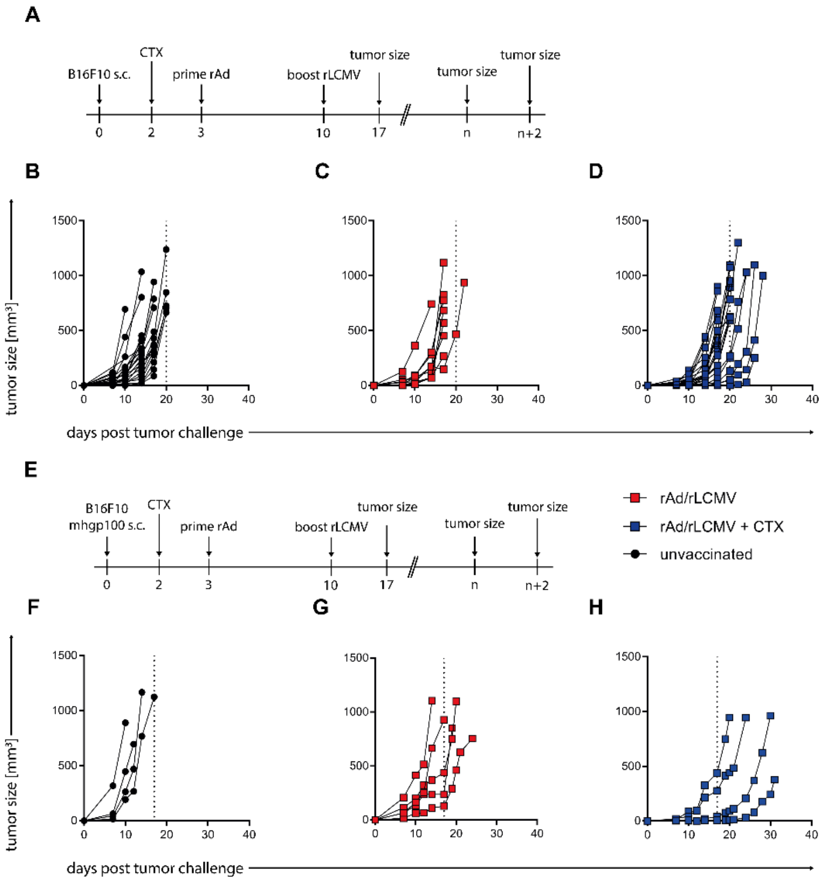


**Supplement Figure 2: Therapeutic immunization with hgp100 against mhgp100 expressing melanoma is enhanced by CTX. A** Experimental setup for therapeutic PB immunization of C57BL/6 mice. Mice were inoculated s.c. with $1x10^5$ cells B16.F10. After two days mice were treated i.p. with CTX. At day 3 mice were subcutaneously prime-immunized with $1x10^9$ physical particles rAd-hgp100. At day 10 mice received a boost-vaccination with rLCMV-hgp100 **B** Tumor growth kinetics in

unvaccinated mice, (**C**) prime boost immunized with rAd/rLCMV-hgp100 (**D**) or rAd/rLCMV-hgp100 in combination with CTX. Data was pooled from four independent experiments with n = 5-6 mice per group. **E** Experimental setup for therapeutic PB immunization of C57BL/6 mice inoculated s.c. with $1x10^5$ cells B16.F10-mhgp100. After two days mice were treated i.p. with CTX. At day 3 mice were subcutaneously prime-immunized with $1x10^9$ physical particles rAd-hgp100. At day 10 mice received a boost-vaccination with rLCMV-hgp100 **F** Tumor growth kinetics in unvaccinated mice, (**G**) prime boost immunized with rAd/rLCMV-hgp100 (**H**) or rAd/rLCMV-hgp100 in combination with CTX (n = 4-5 mice per group). **P* < 0.5, ***P* < 0.01, ****P* < 0.001.

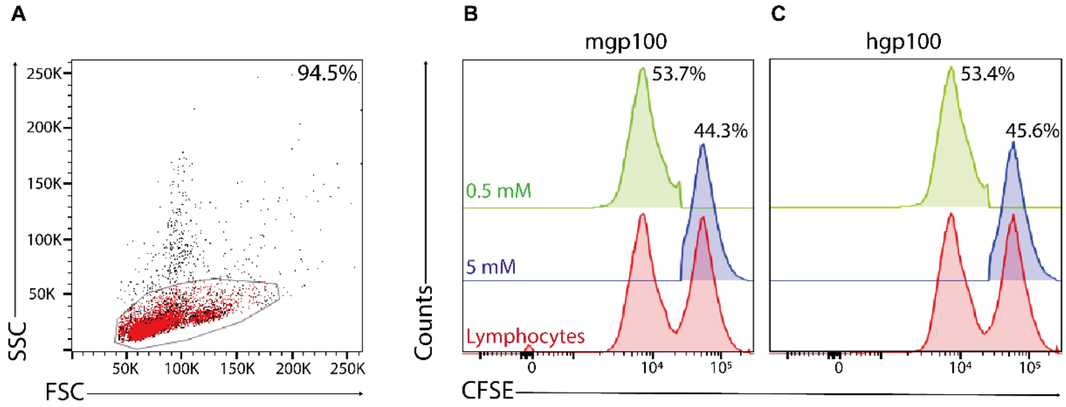


**Supplement Fig. 3. Heterologous prime boost immunization induces CD8+ T cells with *in vivo* killing capacity against gp100 pulsed splenocytes. A** Representative FACS plot of CFSE labelled splenocytes before intravenous injection into mice. Splenocytes were incubated with $10^{-6}$ M (**B**) $mgp100_{25-33}$ or (**C**) $hgp100_{25-33}$ peptide and labelled with 0.5 mM (unpulsed) or 5 mM (pulsed) CFSE. 24 hours later the CFSE expression in peripheral blood was analyzed and the specific killing of peptide pulsed cells determined. Data was pooled from two independent experiments with n = 3-4 mice per group. **P* < 0.5, ***P* < 0.01, ****P* < 0.001.
